# Conservative orthodontic-surgical management of a mandibular premolar impaction associated with a dentigerous cyst: A case report

**DOI:** 10.1097/MD.0000000000046786

**Published:** 2025-12-19

**Authors:** Viet Anh Nguyen, Thi Quynh Phuong Vo

**Affiliations:** a Faculty of Dentistry, Phenikaa University, Hanoi, Vietnam; b Department of Orthodontics, Viet Anh Orthodontic Clinic, Hanoi, Vietnam.

**Keywords:** conservative treatment, dentigerous cyst, impacted tooth, mandibular premolar, orthodontic traction

## Abstract

**Rationale:**

Dentigerous cysts are the second most common odontogenic cysts and are frequently associated with impacted permanent teeth. Conventional management typically involves enucleation with extraction of the involved tooth, which often leads to tooth loss and alveolar bone resorption. This case illustrates a conservative surgical orthodontic protocol that preserved the impacted tooth, maintained alveolar bone support, and corrected the malocclusion.

**Patient concerns:**

A 15-year-old male presented with the chief complaint of dental crowding and facial asymmetry. Clinical examination revealed mandibular deviation to the right, a canted occlusal plane, and smile asymmetry. Intraorally, he exhibited 27 permanent teeth, with retained primary molars and an unerupted mandibular left first premolar.

**Diagnoses:**

The patient was diagnosed with a mild skeletal Class II malocclusion due to maxillary protrusion, mandibular asymmetry, a canted occlusal plane, slight crowding in both arches, and an impacted mandibular left first premolar associated with a dentigerous cyst.

**Interventions:**

Treatment consisted of staged decompression followed by cyst enucleation, surgical exposure of the impacted premolar, and orthodontic traction using a lingual appliance system. The impacted premolar successfully erupted into the dental arch within 5 months.

**Outcomes:**

Comprehensive orthodontic treatment over a 22-month period achieved proper alignment, functional occlusion, and improved smile esthetics. Despite mild root shortening of the premolar, periodontal health, bone support, and tooth stability were preserved at follow-up.

**Lessons:**

A biologically conservative protocol combining cyst management and guided orthodontic traction can effectively manage dentigerous cyst-associated impactions while preserving the natural dentition and avoiding prosthetic replacement.

## 1. Introduction

Dentigerous cysts are the second most common type of odontogenic cyst, most frequently associated with impacted permanent teeth in the mandibular premolar and molar regions.^[1–3]^ While enucleation or marsupialization alone has long been regarded as the standard treatment, the concomitant management of impacted teeth within the cystic cavity remains a unique clinical challenge.^[4]^ Increasing evidence supports a conservative approach combining surgical decompression or enucleation with orthodontic traction, with the goals of preserving the natural dentition, maintaining alveolar bone integrity, and optimizing both functional and esthetic outcomes.^[5,6]^

The present case illustrates such a protocol, in which a mandibular premolar impacted within a cystic lesion was managed through initial decompression, followed by enucleation, surgical exposure, and controlled orthodontic traction with a lingual appliance system. This biologically conservative approach not only preserved the tooth and its alveolar support but also reestablished functional occlusion and enhanced smile esthetics, avoiding the need for prosthetic replacement or orthognathic intervention. Moreover, this report underscores the clinical feasibility and applicability of managing cyst-associated impactions through a biologically conservative, esthetically driven strategy.

## 2. Case presentation

### 2.1. Diagnosis and etiology

**Figure 1.**
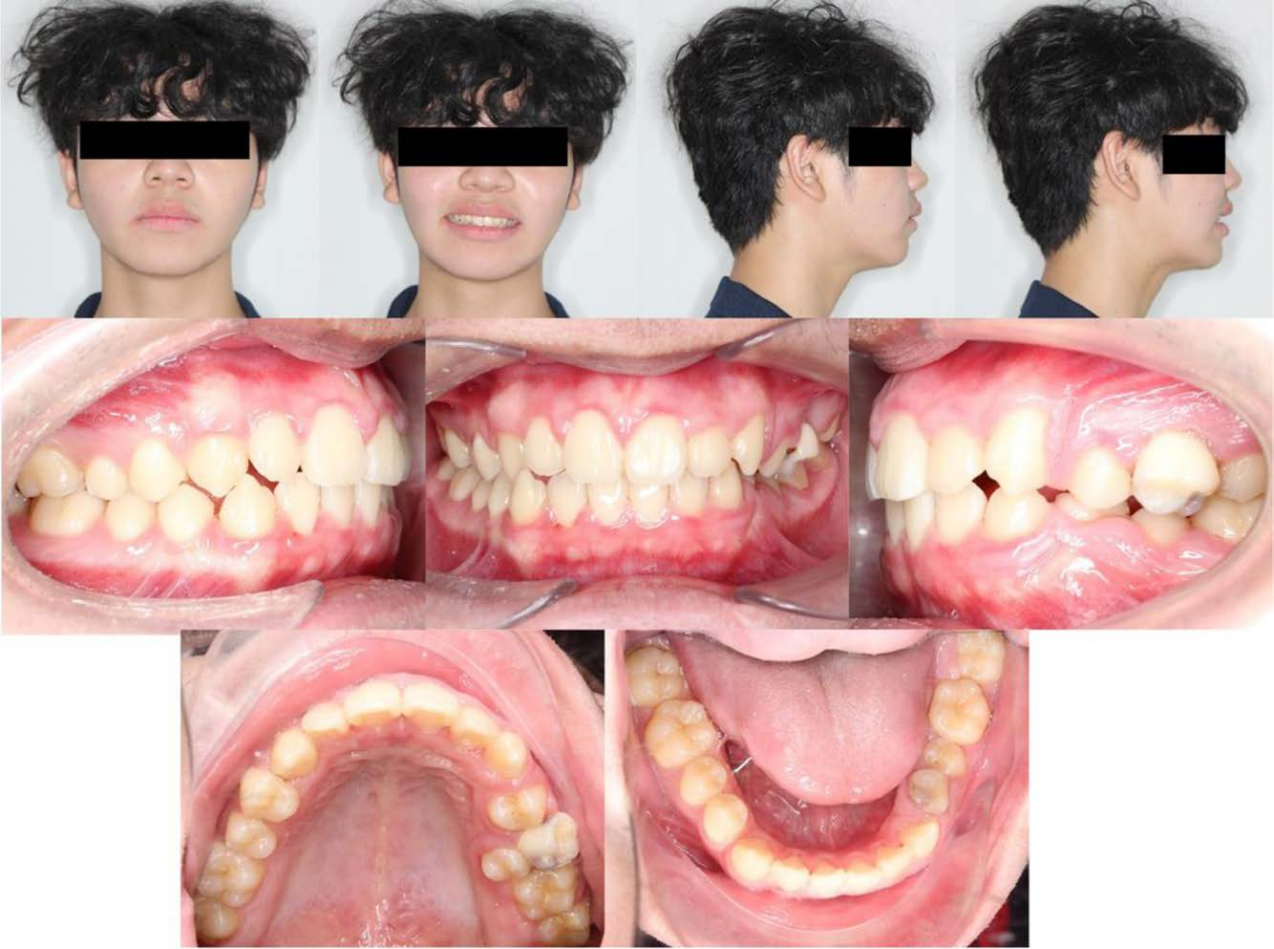
Pretreatment extraoral and intraoral photographs.

A 15-year-old male patient presented with the chief complaint of dental crowding and facial asymmetry. Extraoral examination revealed a convex facial profile with mandibular deviation to the right, a medium smile line, a consonant smile arc, and a canted occlusal plane with inclination toward the right side (Fig. 1). Smile analysis demonstrated marked asymmetry, characterized by elevation of the right oral commissure relative to the left, producing an oblique curvature of the lip line upon smiling.

Intraorally, the patient exhibited 27 permanent teeth, with congenital absence of the mandibular left first premolar. Both the maxillary left second primary molar and mandibular left first primary molar remained in situ. Moderate spacing was observed in the maxillary arch, predominantly on the left side, where the maxillary left second premolar was ectopically erupted toward the palatal aspect. In the mandibular arch, 1 mm of spacing was noted distal to each canine, while the mandibular left second premolar was partially erupted. A deep curve of Spee was evident in the mandibular arch. The maxillary dental midline was shifted 1 mm to the right. The patient exhibited an overjet of 3 mm, an overbite of 2 mm, and an angle Class I molar relationship, except for a unilateral Class II canine relationship on the left side.

**Figure 2.**
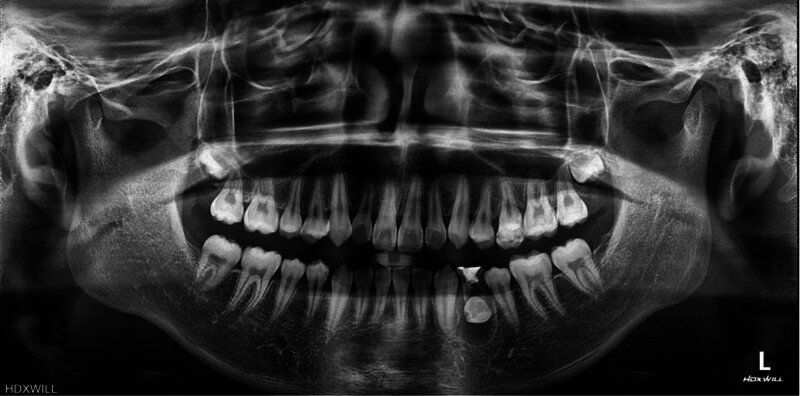
Pretreatment panoramic radiograph.

A panoramic radiograph revealed mandibular skeletal asymmetry between the right and left sides, together with mild alveolar bone crest resorption in the posterior regions of both arches. The mandibular left first premolar was impacted, positioned adjacent to the root of the mandibular left first primary molar, and surrounded by a well-defined pericoronal radiolucency (Fig. 2).

**Figure 3.**
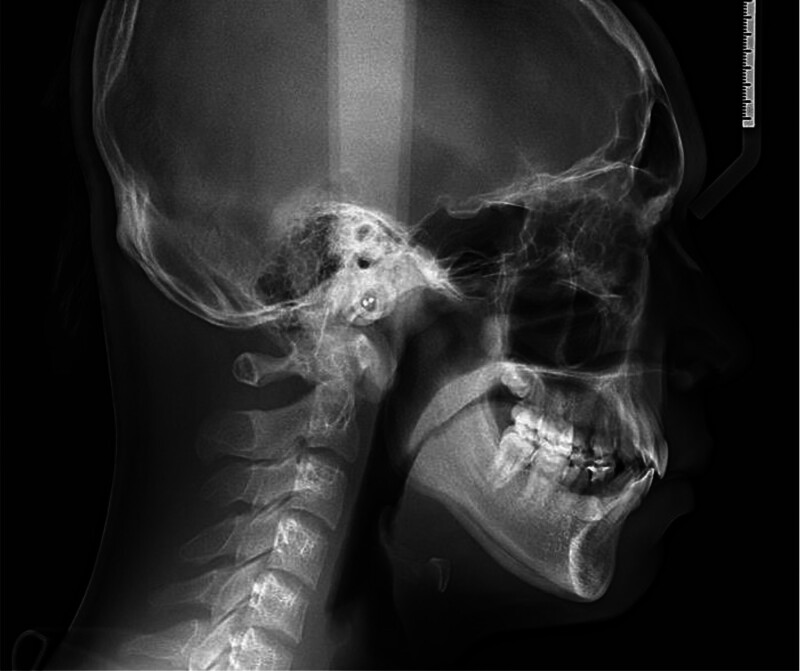
Pretreatment cephalometric radiograph.

Cephalometric (Fig. 3) analysis indicated a prognathic maxilla (SNA, 85.26°) relative to the cranial base and a normally positioned mandible (SNB, 80.59°), resulting in a skeletal Class II relationship (ANB, 4.67°) due to maxillary protrusion (Table 1). The vertical skeletal pattern was within normal limits. Dentally, the maxillary incisors were normally inclined and positioned (U1-SN, 104.81°; U1-NA, 19.55°, 3.98 mm), whereas the mandibular incisors were proclined (IMPA, 94.53°; L1-NB, 29.88°, 8.03 mm), contributing to a reduced interincisal angle (125.90°). The overjet and overbite measurements were within normal limits.

**Table 1:** Lateral cephalometric measurements.

Measurements	Pretreatment	Posttreatment	Norm
Skeletal			
SNA (°)	85.26	85.02	81.1 ± 3.7
SNB (°)	80.59	81.14	79.2 ± 3.8
ANB (°)	4.67	3.88	2.5 ± 1.8
FMA (°)	24.40	23.87	25.0 ± 4.0
Wits appraisal (mm)	1.80	0.3	0.4 ± 2.3
Dental			
Upper incisor/SN (°)	104.81	106.39	105.3 ± 6.6
Upper incisor/NA (°)	19.55	22.68	22.0 ± 5.0
Upper incisor/NA (mm)	3.98	5.42	4.0 ± 3.0
IMPA (°)	94.53	93.2	90.0 ± 3.5
Lower incisor/NB (°)	29.88	27.35	25.0 ± 5.0
Lower incisor/NB (mm)	8.03	7.2	4.0 ± 2.0
Interincisal angle (°)	125.90	127.4	128.0 ± 5.3
Overjet (mm)	2	1.5	2.0 ± 2.0
Overbite (mm)	2	1.3	2.0 ± 2.0
Soft tissue			
Upper lip/E-line (mm)	1.92	1.52	0.0 ± 2.0
Lower lip/E-line (mm)	3.72	1.15	0.0 ± 2.0

ANB = A point–Nasion–B point, FMA = Frankfort mandibular angle, IMPA = incisor mandibular plane angle, NA = Nasion-point A, NB = Nasion-point B, SN = Sella-Nasion, SNA = Sella-Nasion-point A, SNB = Sella-Nasion-point B.

Soft tissue analysis revealed that both the upper lip (1.92 mm) and lower lip (3.72 mm) were positioned anterior to Ricketts’ esthetic line, producing a mildly protrusive soft tissue profile.

**Figure 4.**
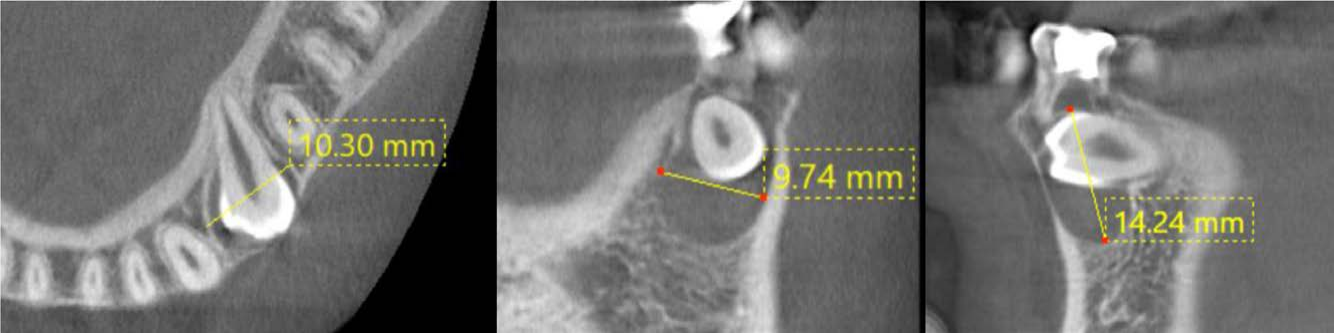
Pretreatment computed tomography image of the mandibular left first premolar.

On the cone-beam computed tomography (CTCB), the mandibular first premolar is horizontally impacted in a buccolingual direction, located immediately beneath its corresponding primary molar. The occlusal surface is approximately 2 mm from the buccal cortical plate of the mandible, while the root lies adjacent to the lingual cortical plate. A radiolucent lesion completely envelops the crown, measuring 10 × 10 × 14 mm (Fig. 4), and is closely associated with the buccal cortical plate.

The patient was diagnosed with a mild skeletal Class II malocclusion characterized by mandibular asymmetry, slight crowding in both arches, a canted occlusal plane, an impacted mandibular left first premolar with an associated dentigerous cyst, and a convex soft tissue profile with bimaxillary lip protrusion relative to the E-line.

### 2.2. Treatment objectives

The foremost treatment objective was to orthodontically extrude the mandibular left first premolar, which was impacted within the alveolar bone, to bring it into functional occlusion. Successful alignment and eruption of this tooth would eliminate the need for prosthetic replacement and preserve the patient’s natural dentition, thereby maintaining alveolar bone volume and long-term periodontal support in the affected area. The plan considered the careful use of controlled traction mechanics to minimize root resorption risk and to maintain periodontal health during the eruption process.

A second objective was to correct the ectopic position of the maxillary left second premolar and to align it within the dental arch. Achieving proper alignment in both the maxillary and mandibular arches was necessary to establish ideal arch coordination and stable interdigitation. This included relieving crowding, coordinating arch widths, and ensuring proper buccolingual inclination of posterior teeth to optimize functional occlusion.

Correction of the dental midline discrepancy was another primary goal. The maxillary and mandibular midlines were planned to be coordinated so that they coincided with each other and were positioned in harmony with the facial midline. This alignment was expected to improve the overall occlusal symmetry and to facilitate optimal intercuspation of the posterior segments.

In addition, improvement of the occlusal plane cant was targeted to enhance dental and smile esthetics. Mechanically, this would be addressed through asymmetric vertical control, selective intrusion and extrusion of posterior teeth, and careful use of anchorage to avoid unwanted occlusal changes. Nevertheless, it was recognized from the outset that the skeletal asymmetry of the mandible would remain uncorrected, as no orthognathic surgical intervention was planned. Consequently, the facial asymmetry and canted smile arc caused by unequal activity of the perioral musculature were not expected to be fully resolved.

Finally, throughout the course of treatment, emphasis was placed on establishing a functional and stable occlusion with ideal overjet and overbite, maintaining periodontal health, and ensuring appropriate axial inclinations of the anterior teeth. The biomechanical plan also aimed to minimize undesirable effects on the patient’s facial soft tissue profile while achieving the occlusal and esthetic goals.

### 2.3. Treatment alternatives

One possible treatment approach was to extract the mandibular left first primary molar along with all 4 first premolars in both arches. This option would eliminate the need for prosthetic replacement of the mandibular left first premolar and avoid the use of miniscrew anchorage. However, excessive retraction of both maxillary and mandibular incisors could compromise facial esthetics and anterior occlusion.

Another alternative was to distalize the maxillary dentition to reduce proclination, extract the mandibular left first primary molar and the impacted mandibular left first premolar, and subsequently replace the missing premolar with a dental implant once the patient reached skeletal maturity. This approach would avoid the extraction of additional permanent teeth and allow better control of incisor inclination. Nevertheless, the requirement for implant placement would add a restorative procedure, increasing treatment time and overall cost.

The third approach involved distalizing the maxillary dentition to reduce proclination, extracting the mandibular left first primary molar, and orthodontically uprighting and aligning the impacted mandibular left first premolar into the arch. This option would maximize preservation of natural dentition, avoid implant placement, and maintain long-term natural function. However, it would require more complex orthodontic mechanics with precise force control to ensure periodontal stability of the previously impacted tooth, especially given its history of pericoronal pathology. This was the treatment plan selected by the patient after being fully informed of the advantages, limitations, and prognosis.

### 2.4. Treatment progress

Prior to the initial digital workflow, the maxillary left second primary molar was extracted to create the necessary space for eruption guidance and precise bracket placement. Bracket positioning was meticulously planned using 3-dimensional orthodontic software, which allowed virtual alignment of the dentition and optimization of the bracket height and angulation according to the individualized prescription.^[7]^ A stereolithographic model was subsequently printed, and a customized transfer tray was fabricated to facilitate indirect bonding. Self-ligating lingual appliances were then bonded to the maxillary and mandibular arches using the indirect bonding protocol, ensuring accurate placement with minimal chairside adjustments. Concurrently, marsupialization of the cystic lesion associated with the mandibular left first premolar was performed to decompress the lesion and promote bone regeneration. Initial alignment and leveling were initiated with a 0.012-inch nickel-titanium (NiTi) archwire in the maxillary arch and a 0.016-inch NiTi archwire in the mandibular arch, allowing for light continuous forces to correct irregularities without jeopardizing periodontal support.

After 1 month, significant reduction in cyst size was noted on radiographic follow-up. The maxillary archwire was sequentially upgraded to 0.014-inch NiTi, while the mandibular archwire was transitioned to 0.016-inch stainless steel (SS) to enhance torque control in preparation for traction mechanics. At this stage, the mandibular left first primary molar was extracted, followed by complete cyst enucleation and surgical exposure of the unerupted mandibular left first premolar. A bonded button attachment was secured to the exposed tooth surface and ligated to the main archwire with a SS ligature tie for passive stabilization during healing. One month after surgical exposure, orthodontic traction was initiated using an open-coil spring assembly, with the mandibular arch maintained on a rigid 0.016 × 0.016-inch SS wire to prevent unwanted arch deformation. The maxillary arch continued progressive alignment with a 0.016-inch NiTi wire.

**Figure 5.**
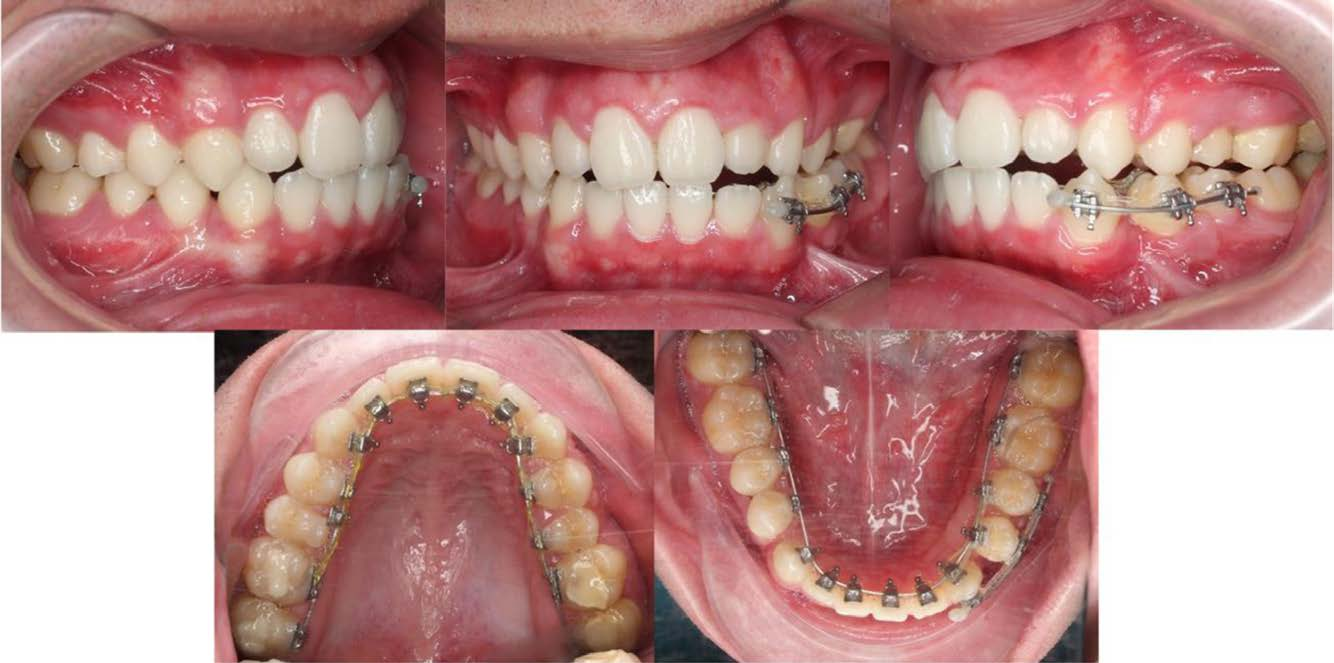
Intraoral and extraoral clinical photographs obtained after 7 mo of treatment.

As the impacted premolar approached the alveolar crest level, a second surgical procedure was carried out to expose the buccal crown surface using an apically positioned flap technique, thereby preserving an adequate band of keratinized gingiva for long-term periodontal health. Buccal preadjusted edgewise brackets were simultaneously bonded to the mandibular left canine and second premolar to facilitate segmental mechanics. The piggyback technique was employed with an auxiliary superelastic NiTi archwire, sequentially progressing from 0.012 to 0.016 and then to 0.016 × 0.022 inches, allowing precise vertical traction of the impacted tooth while maintaining overall arch stability on the lingual system with a 0.016-inch SS main archwire (Fig. 5).

Upon successful alignment of the mandibular left first premolar into the dental arch, a lingual bracket was bonded to integrate the tooth fully into the lingual appliance system. Comprehensive re-leveling of the mandibular arch was undertaken, commencing with a 0.012-inch NiTi archwire and progressing to 0.016-inch NiTi lingually, combined with a 0.019 × 0.025-inch NiTi buccal sectional wire on the left segment for additional torque and angulation control. Mid-treatment evaluation revealed moderate residual spacing in the mandibular arch, an edge-to-edge relationship in the right premolar segment, and a molar relationship consistent with Class II on the right side. To address these discrepancies, intermaxillary cross elastics and Class II elastics were prescribed, supplemented by continuous power chain application for mandibular space closure.

**Figure 6.**
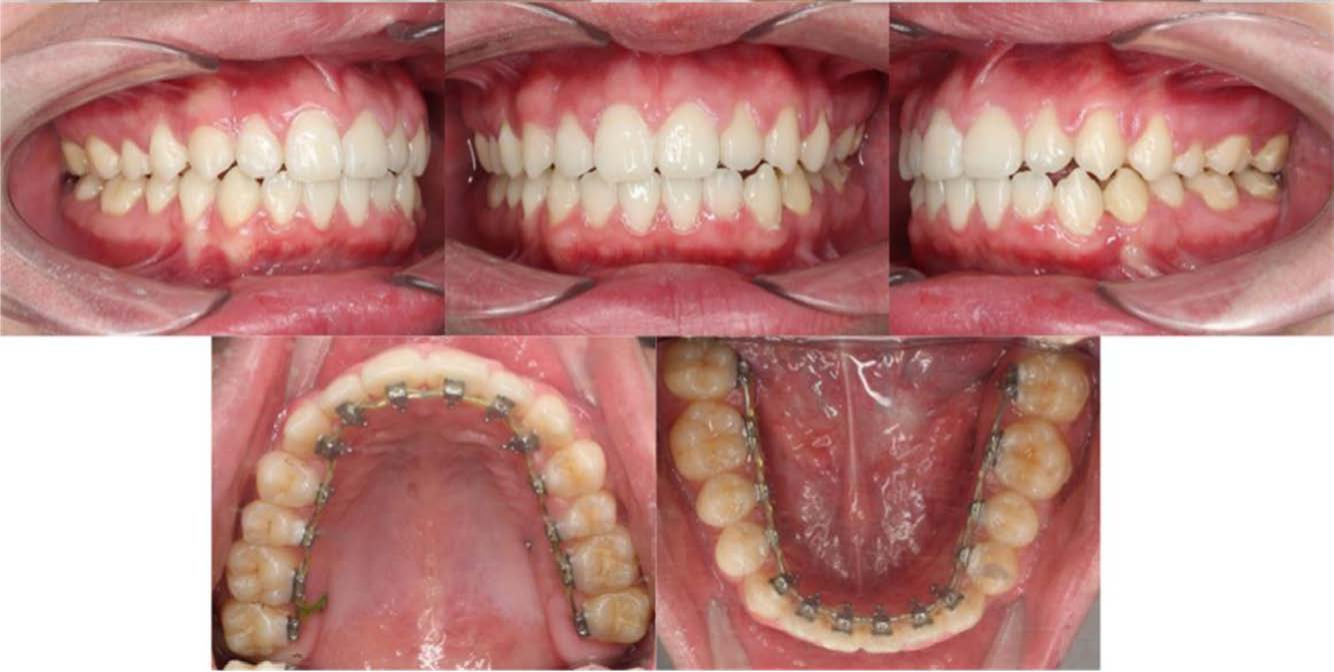
Intraoral and extraoral clinical photographs obtained after 20 mo of treatment.

At 9 months into active treatment, both arches were engaged with 0.016 × 0.022-inch SS archwires to facilitate final torque expression. Positive labial crown torque was incorporated into the maxillary anterior segment to improve incisor inclination and compensate for previous anchorage demands. Two temporary anchorage devices (1.6 mm diameter, 10 mm length) were strategically inserted in the palatal alveolar bone – one between the maxillary right first and second molars, and the other between the maxillary left second premolar and first molar – to serve as direct anchorage units for en-masse maxillary distalization. Concurrently, mandibular space closure was continued with elastomeric chains. Two months later, a torque-adjusted mandibular SS archwire was implemented to counteract lingual tipping of the anterior teeth (Fig. 6) that had occurred during space closure. Intermaxillary elastics were maintained to refine intercuspation and achieve optimal cusp-to-fossa relationships. After approximately twelve months of space closure, the occlusion exhibited complete space consolidation, stable intercuspation, and proper sagittal correction.

**Figure 7.**
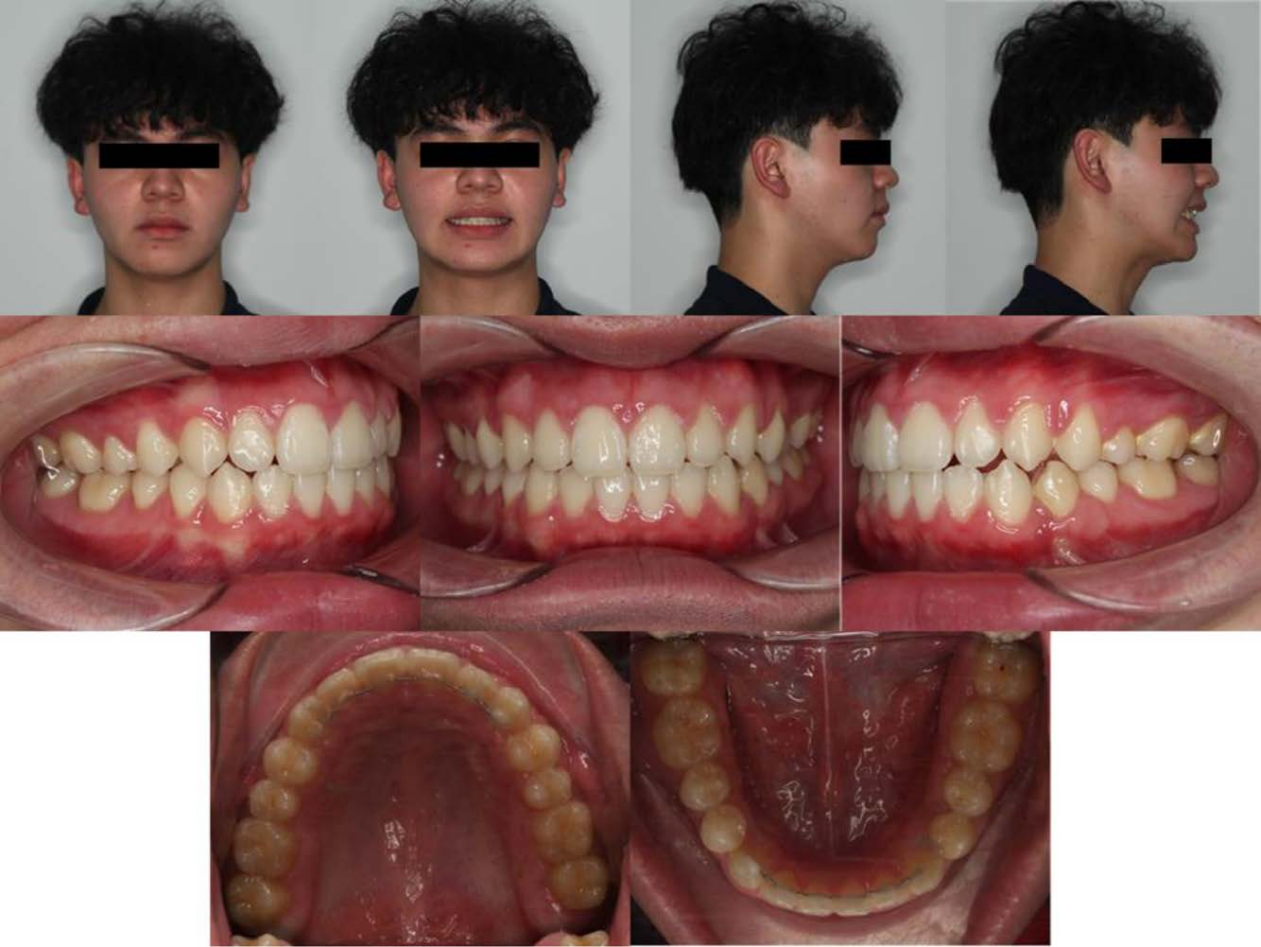
Intraoral and extraoral photographs after treatment.

The total active treatment duration was 22 months, comprising 2 months of initial alignment and leveling, 5 months of impacted premolar traction, 12 months of space closure, and 3 months of finishing and detailing. Following appliance removal, fixed lingual retainers were bonded to the 6 anterior teeth in both arches (Fig. 7), complemented by vacuum-formed clear retainers to ensure long-term stability.

### 2.5. Treatment results

Posttreatment records demonstrated marked improvement in the patient’s facial profile. The upper and lower lips were retracted relative to Ricketts’ esthetic line, producing a more balanced and harmonious soft tissue profile. Nevertheless, a residual mandibular deviation to the right persisted due to the underlying skeletal asymmetry. Smile esthetics improved substantially, with correction of the occlusal plane cant contributing to a more symmetric smile display.

Intraorally, the dentition was well aligned, with stable intercuspation and bilateral Class I canine and molar relationships. The maxillary and mandibular midlines were coincident with each other and with the facial midline. The curve of Spee was leveled, and the overjet (1.5 mm) and overbite (1.3 mm) were maintained within normal limits.

**Figure 8.**
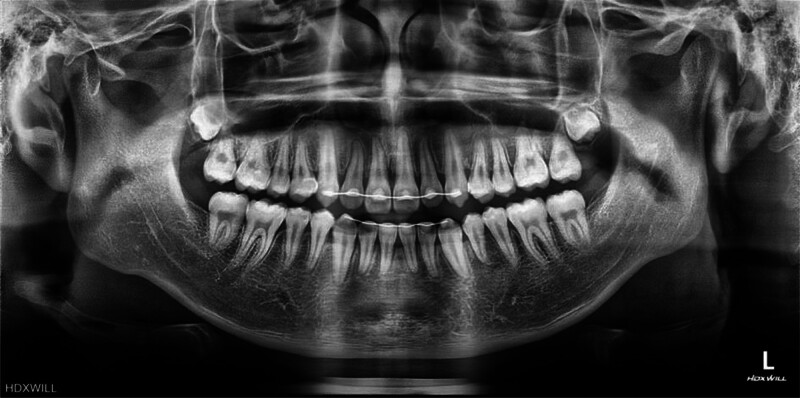
posttreatment panoramic radiograph.

The posttreatment panoramic radiograph revealed no additional alveolar bone loss compared with pretreatment findings. The previously impacted mandibular left first premolar was successfully aligned into the arch with upright root angulation and homogeneous alveolar bone density surrounding the root (Fig. 8). However, shortening of the root resulted in a crown-to-root ratio approximating 1:1, necessitating periodic radiographic monitoring.

**Figure 9.**
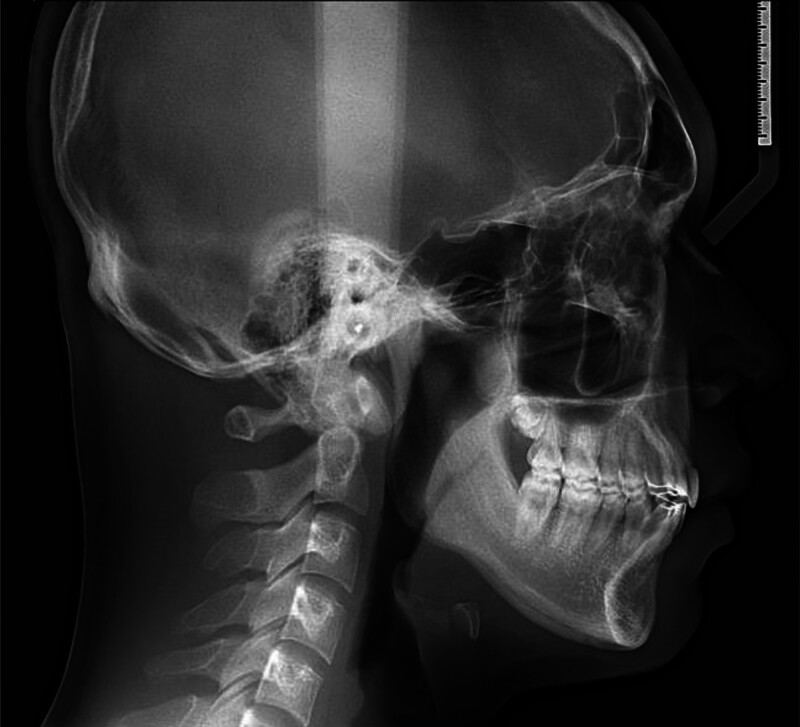
posttreatment cephalometric radiograph.

Cephalometric analysis demonstrated skeletal and dental changes consistent with the treatment objectives (Fig. 9). The maxillary showed a slight decrease in SNA (85.26°–85.02°), primarily attributable to retraction of the maxillary incisors. The mandible advanced modestly (SNB, 80.59°–81.14°), reducing the skeletal discrepancy as reflected in the decrease of ANB from 4.67° to 3.88°. The vertical skeletal relationship remained stable (FMA, 24.40°–23.87°), and the Wits appraisal improved from 1.80 to 0.3 mm, approaching the normative value.

Dentally, the maxillary incisors maintained favorable inclination (U1-SN, 104.81°–106.39°; U1-NA, 19.55°–22.68°) with increased retraction relative to NA (3.98–5.42 mm). The mandibular incisors were uprighted and retracted (IMPA, 94.53°–93.2°; L1-NB, 29.88°–27.35°; L1-NB linear, 8.03–7.2 mm), resulting in an increased interincisal angle (125.9°–127.4°).

Soft tissue evaluation revealed favorable changes. The upper lip was retracted slightly (1.92–1.52 mm anterior to the E-line), while the lower lip demonstrated a more pronounced improvement (3.72–1.15 mm), yielding enhanced lip competence and a more esthetically balanced profile.

Overall, posttreatment cephalometric values approached the normative ranges, particularly for skeletal parameters (ANB, Wits appraisal), incisor inclinations, and lip positions. These changes reflect the achievement of not only optimal occlusal function and alignment but also a harmonious facial esthetic outcome.

## 3. Discucssion

This case illustrates that a comprehensive orthodontic protocol combining cyst decompression, surgical exposure, controlled traction mechanics, and a lingual appliance system can effectively manage impacted teeth associated with large dentigerous cysts. By preserving the natural dentition, establishing functional occlusion, and improving smile esthetics without the need for prosthetic replacement, this treatment approach underscores the advantages of a biologically conservative and esthetically driven strategy.

In managing this case, extraction-based protocols or implant replacement of the impacted premolar were theoretical alternatives; however, both carried notable drawbacks. Multiple premolar extractions would likely have led to excessive anterior retraction, flattening of the profile, and compromised smile esthetics, as previously emphasized by Baik et al.^[8]^ Implant rehabilitation in an adolescent is also contraindicated due to continued craniofacial growth and the risk of infraocclusion, as highlighted by Komisarek et al.^[9]^ By comparison, for mandibular third molars involved in large cystic lesions, extraction of the tooth together with enucleation of the associated lesion is often considered appropriate and definitive, because these posterior teeth are frequently nonessential for functional occlusion and are more difficult to reposition orthodontically.^[10]^ In contrast, the chosen approach – orthodontic traction of the impacted premolar following cyst decompression and surgical exposure – was biologically conservative, preserved alveolar bone and periodontal support, and provided superior long-term functional and esthetic outcomes, consistent with reports by El-Beialy et al.^[5]^

In adult and late-adolescent cases of impacted teeth associated with dentigerous cysts, a growing body of evidence supports conservative management combining surgical decompression (marsupialization or enucleation) with controlled orthodontic traction to preserve natural dentition and encourage bone regeneration. Chung et al reported the successful eruption of 2 mandibular molars impacted within a large expansile cyst through marsupialization followed by traction using skeletal anchorage (miniplates), achieving results over a 4-year treatment period in an adolescent/adult context.^[4]^ Tsironi et al described uprighting a deeply impacted mandibular first molar with concomitant dentigerous cyst through combined surgical and orthodontic treatment – after 18 months of traction, new bone formed and functional occlusion was attained.^[11]^ Celebi et al also documented successful orthodontic repositioning of an impacted mandibular third molar within a cyst following marsupialization.^[12]^

These clinical observations are corroborated by systematic data. Nahajowski and colleagues’ meta-analysis found that while a proportion of teeth may erupt spontaneously post-marsupialization, many require orthodontic guidance; in adult presentations where spontaneous eruption is less predictable, the controlled application of traction becomes essential.^[13]^ Maltoni et al demonstrated a fixed orthodontic and surgical hybrid approach to rescue 2 impacted teeth enmeshed in a large cyst via controlled biomechanics.^[14]^ An interdiciplinary case using modified appliances (like a decompression lingual arch) also reinforced that innovative mechanics can guide eruption safely in adults.^[15]^ Wei et al reported on adult management where the lesion and tooth were large and complex enough to necessitate surgical extraction rather than orthodontic rescue, highlighting factors limiting conservative options in mature patients.^[4,16]^

Biomechanical principles emphasized across these studies consistently include the use of light, continuous forces, anchorage reinforcement, and staged traction mechanics to minimize risks of root resorption or periodontal damage. For instance, Tsironi et al emphasized slow uprighting with attention to bone deposition around the cusp of traction^[11]^; similarly, Maltoni report utilized fixed appliance support to direct eruptive vectors precisely.^[14]^ These approaches parallel the method in our case: after enucleation, an open-coil spring, rigid base archwire, and piggyback NiTi archwire provided controlled upward force to the premolar, preserving bone and avoiding untoward movement – consistent with best practices shown in literature.

In conclusion, the described case aligns well with the clinical and biomechanical strategies supported for adult patients: cyst decompression or removal, followed by carefully staged orthodontic traction utilizing light forces and stable anchorage, can successfully achieve eruption – even in dense mandibular bone contexts. The observed root shortening in our case underscores the importance of long-term radiographic monitoring – a common theme across adult case experiences. Collectively, these findings affirm that conservative surgical orthodontic protocols remain viable, effective, and predictable for managing dentigerous cyst-associated impactions in mature individuals.

This case report presents the outcome of a single patient and, therefore, cannot be readily generalized to broader populations without caution. Although the treatment protocol achieved successful eruption and alignment of the impacted mandibular premolar, several limitations remain. Notably, the shortened root length of the erupted tooth – likely influenced by prolonged impaction and the application of orthodontic traction – raises concerns regarding long-term stability and periodontal prognosis. Furthermore, no histopathological examination of the enucleated cystic tissue was performed, and the diagnosis was based solely on radiographic interpretation, which may limit diagnostic precision. Finally, although follow-up radiographs demonstrated satisfactory bone regeneration and stable occlusion, an extended observation period would be necessary to confirm the long-term viability of the erupted tooth and to monitor for potential recurrence or complications. Future studies or case series involving larger samples and standardized biomechanical protocols would be valuable to validate and refine this conservative approach.

## 4. Conclusion

This case highlights that a biologically conservative protocol combining cyst enucleation, surgical exposure, and controlled orthodontic traction can successfully manage dentigerous cyst-associated impactions even in the mandibular premolar region. By preserving the natural tooth, maintaining alveolar bone integrity, and achieving functional occlusion and esthetic improvement without the need for prosthetic replacement or orthognathic surgery, this approach demonstrates the clinical value of interdisciplinary, well-planned biomechanics. Long-term follow-up remains essential to monitor root stability and periodontal health, but the outcome underscores that conservative surgical orthodontic management is a viable and predictable treatment option for similar complex cases.

## Author contributions

**Conceptualization:** Viet Anh Nguyen.

**Data curation:** Viet Anh Nguyen.

**Formal analysis:** Viet Anh Nguyen.

**Funding acquisition:** Viet Anh Nguyen.

**Investigation:** Thi Quynh Phuong Vo.

**Methodology:** Viet Anh Nguyen.

**Project administration:** Viet Anh Nguyen.

**Resources:** Viet Anh Nguyen.

**Software:** Viet Anh Nguyen.

**Supervision:** Viet Anh Nguyen.

**Validation:** Viet Anh Nguyen.

**Visualization:** Thi Quynh Phuong Vo.

**Writing – original draft:** Thi Quynh Phuong Vo.

**Writing – review & editing:** Viet Anh Nguyen.
